# Genomic Characterisation of a Highly Divergent Siadenovirus (Psittacine Siadenovirus F) from the Critically Endangered Orange-Bellied Parrot (*Neophema chrysogaster*)

**DOI:** 10.3390/v13091714

**Published:** 2021-08-28

**Authors:** Ajani Athukorala, David N. Phalen, Ashutosh Das, Karla J. Helbig, Jade K. Forwood, Subir Sarker

**Affiliations:** 1 Department of Physiology, Anatomy and Microbiology, School of Life Sciences, La Trobe University, Melbourne, VIC 3086, Australia; a.athukorala@latrobe.edu.au (A.A.); k.helbig@latrobe.edu.au (K.J.H.); 2 Sydney School of Veterinary Science, University of Sydney, Camden, NSW 2570, Australia; david.phalen@sydney.edu.au; 3 Schubot Exotic Bird Health, Texas A&M College of Veterinary Medicine and Biomedical Sciences, College Station, TX 77843, USA; 4 Research School of Biology, The Australian National University, 34 Linnaeus Way, Acton, Canberra, ACT 2601, Australia; Ashutosh.Das@anu.edu.au; 5 Department of Genetics and Animal Breeding, Chattogram Veterinary and Animal Sciences University, Zakir Hossain Road, Khulshi, Chattogram 4225, Bangladesh; 6 School of Biomedical Sciences, Charles Sturt University, Wagga Wagga, NSW 2678, Australia; jforwood@csu.edu.au

**Keywords:** *Adenoviridae*, siadenovirus, psittacine siadenovirus *F*, next-generation sequencing, evolution, orange-bellied parrot

## Abstract

Siadenoviruses have been detected in wild and captive birds worldwide. Only nine siadenoviruses have been fully sequenced; however, partial sequences for 30 others, many of these from wild Australian birds, are also described. Some siadenoviruses, e.g., the turkey siadenovirus A, can cause disease; however, most cause subclinical infections. An example of a siadenovirus causing predominately subclinical infections is psittacine siadenovirus 2, proposed name psittacine siadenovirus F (PsSiAdV-F), which is enzootic in the captive breeding population of the critically endangered orange-bellied parrot (OBP, *Neophema chrysogaster*). Here, we have fully characterised PsSiAdV-F from an OBP. The PsSiAdV-F genome is 25,392 bp in length and contained 25 putative genes. The genome architecture of PsSiAdV-F exhibited characteristics similar to members within the genus *Siadenovirus*; however, the novel PsSiAdV-F genome was highly divergent, showing highest and lowest sequence similarity to skua siadenovirus A (57.1%) and psittacine siadenovirus D (31.1%), respectively. Subsequent phylogenetic analyses of the novel PsSiAdV-F genome positioned the virus into a phylogenetically distinct sub-clade with all other siadenoviruses and did not show any obvious close evolutionary relationship. Importantly, the resulted tress continually demonstrated that novel PsSiAdV-F evolved prior to all known members except the frog siadenovirus A in the evolution and possibly the ancestor of the avian siadenoviruses. To date, PsSiAdV-F has not been detected in wild parrots, so further studies screening PsSiAdV-F in wild Australian parrots and generating whole genome sequences of siadenoviruses of Australian native passerine species is recommended to fill the siadenovirus evolutionary gaps.

## 1. Introduction

The genus *Siadenovirus* is one of six adenoviruses families (reviewed in Harrach et al. [1,2]). The basal clade in the *Siadenovirus* genus contains two viruses, frog siadenovirus A detected in the cell culture of a renal tumour from the northern leopard frog (*Lithobates pipiens*) [3], and the Sulawasi tortoise siadenovirus 1 detected in diseased tissues from smuggled impressed tortoises (*Manouria impressa*) and a Burmese star tortoise (*Geochelone platynota*) [4]. Therefore, the *Siadenovirus* genus may have originated in amphibians or reptiles [3,4], although this is still speculative [1]; all other siadenoviruses have been detected in birds [5].

Avian siadenoviruses have been detected in wild birds from three continents—Antarctic [6,7], Australia [5,8], and Europe [9]—and in captive birds originating from Africa [9,10], Asia [10,11], the Indo-Pacific [10,11], including Australia [12,13], and South America [10,14]. They have evolved to infect a wide range of birds and have been detected in birds from the orders Columbiformes [5,15], Charadriiformes [7], Falconiformes [16], Galliformes [17], Passeriformes [9,12,18], Podargiformes [5], Psittaciformes [5,8,10,11,12,13,14], Sphenisciformes [6], and Strigiformes [5]. The majority of the sequence data for avian siadenoviruses is confined to small sequences (approximately 280 bp) of the DNA polymerase gene with full genome sequences limited to only nine unique viruses [3,6,7,8,14,16,17]. This means that only a skeletal phylogram for the avian siadneoviruses can be calculated and the relationships of the avian siadenoviruses with limited sequence data to other siadenovirus can only be approximated [5].

One avian siadenovirus, with limited sequence data, is psittacine siadenovirus 2, which was subsequently referred to as psittacine siadenovirus F (PsSiAdV-F) [11]. The epizootiology and pathogenicity of the PsSiAdV-F is perhaps the best studied of the adenoviruses infecting psittacine birds. It has a wide host range and has been detected in droppings and tissues from psittacine birds originating from their Pacific, Afro-Asian, and neotropical distributions [10,11,12,13]. The natural host species of this virus is not known, but the virus appears to have disseminated globally as the result of the pet trade in wild caught and captive-raised psittacine birds [10,12]. Most infections are subclinical and in the captive breeding population of the critically endangered orange-bellied parrot (*Neophema chrysogaster*), up to 77% of the birds tested can be shedding virus at any one time. It may also be widespread in other captive collections of psittacine birds [12,13]. It can also cause disease. It appears to have a tropism for collecting ducts in the kidney but may also cause the more classical hepatitis seen in other avian adenovirus infections [12,13]. Additionally, Yang et al. [13] found a negative correlation with the prevalence of infection in aviaries breeding the orange-bellied parrot and the numbers of chicks produced per clutch. However, in general, disease is rare and is likely to occur in psittacine birds that are stressed or have concurrent disease, e.g., aspergillosis, or possibly in naïve species of psittacine birds who have not co-evolved with the virus [12,13].

In this study, we report the entire sequence of PsSiAdV-F. We demonstrated that it forms a monophyletic clade that is basal to all other known avian siadenoviruses, suggesting that the avian siadenoviruses originated in psittacine birds, possibly in Australia.

## 2. Materials and Methods

### 2.1. Source of Sample, Extraction of DNA, and Confirmation of the Presence of PsSiAdV-F DNA

Liver tissue of orange-bellied parrot that died as the result of a *Pseudomonas septicemia* was obtained, and total genomic DNA was extracted using a commercial kit (PurelinkTM Genomic DNA Mini Kit, Invitrogen, Carlsbad, CA, USA) following the manufacturer’s instructions. A widely used PCR protocol capable of detecting all known adenoviruses was performed to screen extracted DNA for a portion of the adenovirus DNA polymerase sequence [13], and an amplicon of expected molecular mass was generated. The amplicon was sequenced in both directions (Australian Genome Research Facility (AGRF), Westmead, NSW, Australia) and was found to be identical to that of the DNA of PsSiAdV-F (data not shown).

### 2.2. Library Construction and Sequencing

The library construction was adapted using the Nextera DNA Flex Prep (Illumina, San Diego, CA, USA) as per kit instructions. The quality and quantity of the prepared library was assessed (AGRF, Westmead, NSW, Australia). The prepared library was normalised and pooled in equimolar quantities. The quality and quantity of the final library was further assessed before sequencing by the AGRF facility. Cluster generation and sequencing of the library was performed with the read length of 150-bp paired-end on Illumina^®^ HiSeq chemistry according to the manufacturer’s instructions.

### 2.3. Genome Assembly

DNA sequencing data were analysed using Geneious (version 20.0.3, Biomatters, Ltd., Auckland, New Zealand) and CLC Genomics Workbench (version 9.5.4, CLC bio, a QIAGEN Company, Prismet, Aarhus C, Denmark) according to the previously established analysing pipeline [8,19,20,21]. The complete genome of PsSiAdV-F was obtained from a total number of 384.8 million paired-end reads. Initial quality evaluation for all raw reads was generated and pre-processed to remove ambiguous base calls and poor-quality reads. Illumina adapter sequences were trimmed, and the trimmed reads were mapped against the chicken genome (*Gallus gallus*, GenBank accession number NC_006088) to remove host DNA contamination. In addition, reads were further mapped to *Escherichia coli* bacterial genomic sequence (GenBank accession no. U00096) to remove possible bacterial contamination. Unmapped reads were subjected to de novo assembly, using SPAdes assembler (version 3.10.1) [22], under the “careful” parameter in LIMS-HPC cluster (La Trobe Institute for Molecular Science—High Performance Computing cluster, specialised for genomics research in La Trobe University) [23,24]. Resulting contigs were compared against the nonredundant nucleotide and protein databases on GenBank using BLASTn and BLASTx [25], respectively, with an e-value threshold of 1 × 10^−5^ to remove potential false positives. BLASTN searches yielded a single contig of 25,392 bp corresponding to a siadenovirus sequence.

### 2.4. Genome Annotation and Bioinformatics

The assembled PsSiAdV-F genome was annotated using the Geneious software package (version 20.0.3, Biomatters, Ltd., Auckland, New Zealand), with psittacine siadenovirus D (PsAdV-D, GenBank accession no. MN687905.1) and turkey siadenovirus A (TAdV-A, GenBank accession no. AC_000016) used as reference genomes. Rather than a single genome, several siadenoviruses genomes were used as references for the annotation process to compare the ORFs of predicted proteins with the genus of *Siadenovirus* and to evaluate the consequences of potential truncations or extensions that can occur at the N- and C-termini of predicted proteins and orthologues. ORFs over 30 amino acids along with minimal overlapping (not exceeding 25% overlaps in one of the genes) to other open reading frames were selected and annotated. The predicted ORFs were extracted into FASTA files subsequently, and similarity searches were performed on annotated ORFs as potential genes to determine whether they shared significant sequence similarities to established viral or cellular genes (BLAST E value ≤ 10^−5^) or contained a putative conserved domain as predicted by protein searches (BLASTX and BLASTP) [26].

In order to predict the function of predicted hypothetical proteins, multiple applications were used to search the derived protein sequence of each ORF and to identify their conserved domains or motifs. TMHMM package v.2.0 (DTU Health Tech, Lyngby, Denmark) [27], Geneious (version 20.0.3, Biomatters, Ltd., Auckland, New Zealand), HMMTOP [28], and TMpred [29] were used to search transmembrane (TM) helices. Conserved secondary structure (HHpred) [30] and protein homologs were searched using Phyre2 [31] and SWISS-MODEL [32] to help predict the function of predicted ORFs in this study.

### 2.5. Comparative Genomics

Organisation of the newly assembled PsSiAdV-F genome with other selected siadenoviruses was visualised and compared, using CLC Genomic Workbench (version 9.5.4, CLC bio, a QIAGEN Company, Prismet, Aarhus C, Denmark). Geneious software (version 20.0.3, Biomatters, Ltd., Auckland, New Zealand) was used to obtain comparative G + C content (%), pairwise identity of representative siadenovirus species against PsSiAdV-F, based on the nucleotide sequences of the complete genome and the similarity percentage of selected siadenoviruses core proteins sequences. Selected proteins were aligned using the alignment algorithm MAFFT, in Geneious (version 20.0.3, Biomatters, Ltd., Auckland, New Zealand), and the similarity percentage of protein sequences was calculated following the scoring matrix BLOSUM62 and Gap open penalty = 1.53. Blosum62 with threshold 1 (percentage of residues that have score > = 1 in the Blosum62 matrix) parameters.

### 2.6. Phylogenetic Analyses

Phylogenetic analysis was performed to determine the evolutionary relationship of the newly assembled PsSiAdV-F genome sequence characterised in this study with 42 other publicly available representative adenovirus genome sequences available in GenBank. Initially, amino acid sequences of four conserved genes—DNA polymerase, pTP, hexon and penton—were extracted individually from the selected AdV genomes. Then, individual and concatenated sequences of the selected genes were separately aligned with MAFTT (version 7.450), using G-INS-I (scoring matrix BLOSUM62; gap open penalty 1.53; off set value 0.123) in Geneious (version 20.0.3, Biomatters, Ltd., Auckland, New Zealand) [33]. Sequences were annotated with the host species followed by AdVs name and GenBank accession number in parentheses. Using the individual and concatenated amino acids sequence alignments, maximum likelihood (ML) [34]-based phylogenetic analyses were performed with 1000 non-parametric bootstrap replicates implemented in CLC Genomics Workbench (version 9.5.4, CLC bio, a QIAGEN Company, Prismet, Aarhus C, Denmark) and Geneious (version 20.0.3, Biomatters, Ltd., Auckland, New Zealand).

### 2.7. Recombination Analyses

Recombination analyses were performed within the siadenoviruses. Full length genome and selected gene sequences of siadenoviruses were assessed for the detection of recombination signals using the RDP, Bootscan, MaxChi, GENECONV, Siscan, Chimaera, LARD, and 3Seq methods contained in the RDP4 program [35]. Events detected with significant *p*-values from at least two of the above-mentioned methods were considered as possible events of recombination.

## 3. Results

### 3.1. Genome of PsSiAdV-F

The assembled PsSiAdV-F complete genome sequenced in this study was a linear double-stranded DNA molecule of 25,392 bp in length, which is the second smallest genome of known siadenoviruses to date. The PsSiAdV-F complete genome from the endangered OBP contained all the conserved coding genes expected within members of the genus *Siadenovirus* and two identical inverted terminal repeats (ITRs) capping the genome. Siadenoviruses have smaller ITRs compared to the other adenoviruses, ranging from 26 to 39 bp [36]. The length of the ITRs in PsSiAdV-F is shorter than other siadenovirus, encompassing 16 bp each with the coordinates of 1–16 sense orientation and 25,377–25,392 antisense orientation. The PsSiAdV-F genome was shown to contain a relatively low G + C percentage (36.9%), which is common among siadenoviruses. Genome analysis revealed that the most closely related AdV genome to the PsSiAdV-F was the skua siadenovirus A (SuAdV-A) with 57.06% genome identity The second most closely related adenovirus was raptor siadenovirus A (55.61%). Other siadenoviruses, except psittacine siadenovirus D and frog adenovirus A, also showed over 50% identity with this PsSiAdV-F genome. Interestingly, psittacine siadenovirus D (GenBank accession no. MN687905) from a closely related host species, budgerigar (*Melopsittacus undulatus*), showed the least genome identity to PsSiAdV-F being 31.1%.

### 3.2. Genome Annotation and Comparative Analyses of PsSiAdV-F

**Figure 1:**
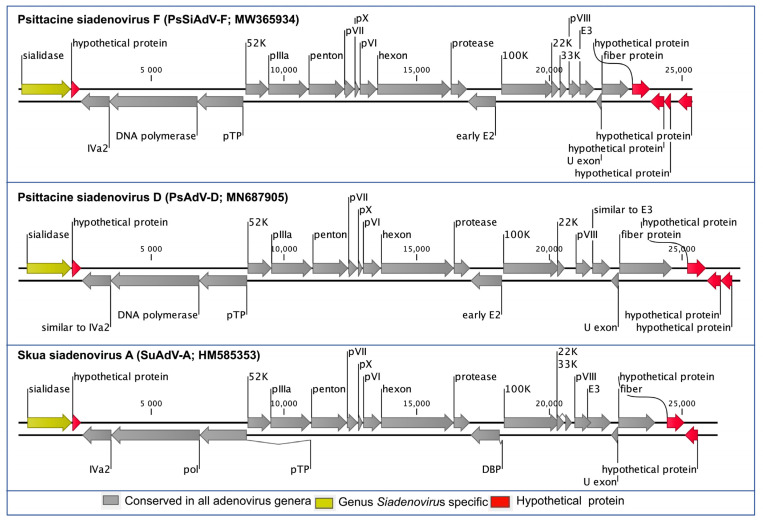
Schematic illustration of the selected siadenoviruses. Schematic map of the psittacine siadenovirus F (PsSiAdV-F, GenBank accession no. MW365934), in comparison with psittacine siadenovirus D (PsAdV-D; GenBank accession no. MN687905) and Skua siadenovirus A (SuAdV-A; GenBank accession no. HM585353) using CLC Genomic Workbench (version 9.5.4, CLC bio, a QIAGEN Company, Prismet, Aarhus C, Denmark). The arrows symbolise adenovirus genes and open reading frames (ORFs) predicted to code for proteins, indicating their direction of transcription. Each gene or ORF is colour coded, as indicated by the colour key in the legend.

The PsSiAdV-F genome encoded 25 predicted methionine-initiated ORFs (numbered from left to right) that were annotated as putative genes (Figure 1 and Table 1). Comparative analysis of the protein sequences encoded by the predicted ORFs, using BLASTX and BLASTP, identified homologs with significant protein sequence similarity (E value: 10^−5^) for given ORFs in Table 1, and according to the BLAST database, no unique genes were identified. Among the predicted 25 protein-coding ORFs of the PsSiAdV-F, 20 were homologous to other siadenovirus common gene products and five were designated as hypothetical proteins (Table 1). Of the five hypothetical proteins, four were identified in the right-hand region of the genome, and one was identified in the left-hand region next to the sialidase gene (Figure 1). Among the 20 homologous protein-coding genes, the highest sequence similarities were observed to psittacine siadenovirus D (PsAdV-D) or turkey siadenovirus A (TAdV-A), at the range of 32% to 73% nucleotide identity in the core genes (Table 1).

The predicted conserved genes in PsSiAdV-F showed the same orientation as SuAdV-A and PsAdV-D, which had the highest and lowest genome identities, respectively (Figure 1). The left-hand region of the PsSiAdV-F genome contained a *Siadenovirus* specific gene homologue of sialidase followed by the gene encoding a homologous hypothetical protein to one found in PsAdV-D and SuAdV-A (Figure 1). The amino acid sequence similarity of sialidase was relatively low compared to the major core genes, with the exception of the fiber gene, which ranged from 34.4% to 44.4% amino acid similarity, with the highest similarity demonstrated with SuAdV-A. Siadenoviruses have a range of hypothetical proteins at their right-hand region. In the right-hand region of PsSiAdV-F, there were four hypothetical proteins, with one on the sense strand and the other three in the antisense strand. Whereas PsAdV-D and SuAdV-A contained three and two hypothetical proteins in this region, respectively (Figure 1).

All the expected adenovirus-conserved genes were present in the center of the PsSiAdV-F genome, and their degree of homology with the other siadenoviruses is presented in Figure 1 and Table 2. The maximum similarity of individual proteins of PsSiAdV-F to homologs in other siadenoviruses varied significantly. As an example, sialidase, DNA polymerase, and DNA-binding protein of PsSiAdV-F displayed the highest pairwise identity with homologous proteins of SuAdV-A, whereas penton and hexon showed the highest identity with raptor siadenovirus A (Table 2). Among the three major capsid proteins, fiber exhibited the lowest amino acid percentage identity (14.06% to 23.82%), whereas hexon and penton showed highest amino acid identity with raptor siadenovirus A (70.98% and 73.79%, respectively). In addition, other predicted conserved proteins including IVa2, pTP, and protease of PsSiAdV-F also demonstrated above 50% identity with their homologous proteins from other siadenovirus species (Table 2).

Additionally, the PsSiAdV-F contained five hypothetical ORFs (ORF-2, -22, -23, -24, and -25, Table 1) that did not show any similarity with known functional proteins in the NR protein database, using BLASTP and BLASTX. These ORFs encoded proteins of 82–225 amino acids (aa) in length (Table 1). Among these, ORF-24 and ORF-25 were shown to contain a single transmembrane helix (TMH) by HMMTOP and TMpred. Nonetheless, there was no evidence for a conserved secondary structure or protein homologs detected by various software, including HHpred [30], Phyre2 [31], and SWISS-MODEL [32] used in this study.

### 3.3. Evolutionary Relationships of PsSiAdV-F

**Figure 2:**
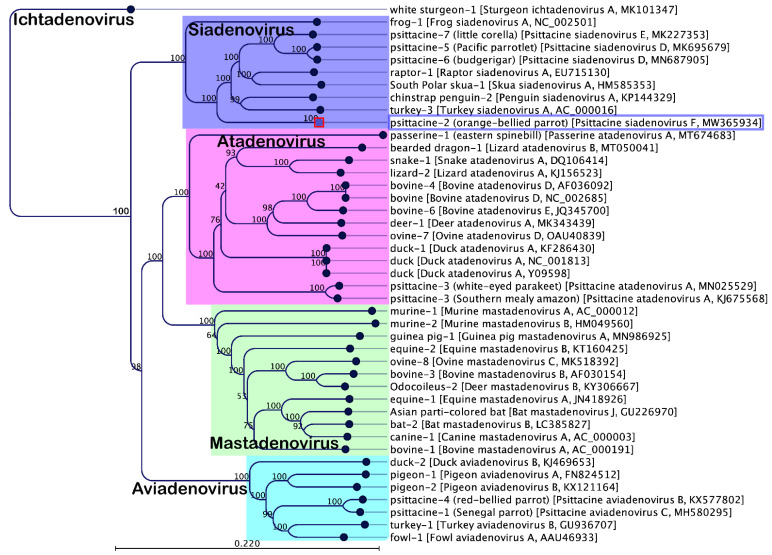
Phylogenetic tree shows the possible evolutionary relationship of novel psittacine siadenovirus F with other selected AdVs. A maximum likelihood (ML) tree was constructed using concatenated amino acid sequences of the complete DNA-dependent DNA polymerase, pTP, penton, and hexon genes. Concatenated protein sequences were aligned with MAFTT (version 7.450) [33] in Geneious (version 20.0.3, Biomatters, Ltd., Auckland, New Zealand) under the BLOSUM62 scoring matrix and gap open penalty = 1.53. The gap > 20 residues were deleted from the alignments. The unrooted ML tree was constructed under the WAG substitution model, and 1000 bootstrap replicates were constructed using tools available in CLC Genomics Workbench (version 9.5.4, CLC bio, a QIAGEN Company, Prismet, Aarhus C, Denmark). The numbers on the left show bootstrap values as percentages, and the labels at branch tips refer to original host species followed by AdVs name and GenBank accession number in parentheses. The novel psittacine siadenovirus F is shown in the purple-coloured box.

Phylogenetic reconstruction based on amino acid sequences of two structural (hexon and penton) and two non-structural (DNA polymerase and pTP) proteins clearly evidences the inclusion of PsSiAdV-F in the genus *Siadenovirus*. PsSiAdV-F occupied a distinct sub-clade in the ML tree generated, based on the concatenated amino acid sequences of these four AdVs genes, with strong bootstrap support (100%) (Figure 2). ML trees based on individual protein sequences of selected DNA polymerase and pTP genes demonstrated a similar tree topology for the PsSiAdV-F species (Supplementary Figures S1 and S2), and all the individual ML trees generated placed PsSiAdV-F into a distinct sub-clade from all other siadenoviruses. This evolutionary relationship of PsSiAdV-F, with no obvious close relationship to other siadenoviruses, evolved from frog adenovirus A (Figure 1 and Supplementary Figures S1–S5), which suggests that it may be an intermediate evolutionary lineage between the basal siadenovirus clade containing frog adenovirus A and the remaining known avian siadenovirues.

### 3.4. Evidence of a Rare Recombination Event

Using the RDP4 program, a recombination event was detected in the DNA polymerase gene of the novel PsSiAdV-F. The support for this recombination was detected in the region spanning from 1173 to 1386 of the DNA polymerase gene of PsSiAdV-F (GenBank accession no. MW365934), where turkey siadenovirus A (GenBank accession no. AC_000016) was a minor and psittacine siadenovirus E was a major (GenBank accession no. MK227353) parental sequence (*p*-value, RDP: 1.49 × 10^−5^, 3Seq: 1.89.86 × 10^−3^). However, there was no further recombination within PsSiAdV-F detected using complete genome or individual gene sequences.

## 4. Discussion

This study describes the first complete genome sequence of the psittacine siadenovirus F (PsSiAdV-F). The sequence was derived from the critically endangered orange-bellied parrot, which is an Australian species. The PsSiAdV-F genome contains all of the major structural and functional gene sequences along with the genus defining the sialidase gene found in other members of the siadenovirus genus. It also has their short-inverted terminal repeats and the characteristically low G + C content characteristic of the siadenoviruses [37]. However, it is highly divergent and clearly represents a separate species, as demonstrated by the low degree of nucleotide and amino acid homology to the closest siadenovirus (Skua siadenovirus A) across its functional and structural genes. Following the International Committee on Taxonomy of Viruses, we propose that this virus be renamed psittacine siadenovirus-F (PsSiAdV-F) (formerly known as psittacine adenovirus 2).

An unexpected finding in the genome of the PsSiAdV-F genome was evidence for a recombination event in the DNA polymerase. Viral recombination plays a key role in the evolutionary mechanisms driving pathogen diversity and host adaptation [38,39,40]. Deep recombination events can also provide evidence for ancient evolutionary relationships, as was shown in a study of an aviadenovirus from a red-bellied parrot (*Poicephalus rufiventris*) [41]. In contrast, this study documents a more recent and rare recombination event among PsSiAdV-F and two other siadenoviruses: turkey siadenovirus A and psittacine siadenovirus E. In viruses, the frequency of recombination and genetic admixture depends on the frequency of co-infections [42], which often acts as the driving force for host-switches and the further emergence of successful pathogens [43,44]. Therefore, we predict that the PsSiAdV-F identified in this study at one or more points in its evolution infected a host that was also infected with turkey siadenovirus A and psittacine siadenovirus E.

PsSiAdV-F in a monophyletic clade that is basal to known avian siadenoviruses suggests that all the avian siadenoviruses evolved from the ancestoral bird that gave rise to PsSiAdV-F. Evidence that this ancestoral species may have been a primitive Australian parrot includes the observation that PsSiAdV-F has only been detected in psittacine species where it causes predominantly subclinical infections and is widespread in captive populations of at least three species of Australian parrot: the orange-bellied parrot, the scarlet-chested parrot (*Neophema splendida*), and the Bourke’s parrot (*Neopsephotus bourkii*) [12,13]. Additionally, there is an extensive diversity of siadenoviruses present in wild and captive native Australian passerine species, and viruses belonging to the more recently evolved siadenovirus clade containing the psittacine siadenovirus E have also been detected in wild Australian psittacine birds [5,8,45]. Ultimate proof that PsSiAdV-F originated in Australian psittacine birds will require that it be identified in a wild Australian psittacine species, and to date, surveys of wild psittacine birds in Australia have not detected it yet [4,7].

The exact phylogenic relationship between the siadneoviruses infecting Australian passerines and OBP remains to be determined. It has been shown previously that atadenoviruses appear to have first arisen in an ancestoral passerine species or possibly in a species prior to the separation of the Passeriformes and Psittaciformes orders [21]. In contrast, our phylogeny, which includes the only full-length DNA polymerase sequence from a passerine (great tit siadenovirus A), suggests that the siadneoviruses infecting passerines evolved subsequent to the establishment of the PsSiAdV-F clade. In an attempt to further investigate the relationship of PsSiAdV-F to the siadenoviruses infecting passerine species, we repeated our analysis including the partial DNA polymerase sequence from the Gouldian siadenovirus B as the virus that has been detected only in the Australian native Gouldian finch (*Chloebia gouldiae*), although only in captive individuals (Supplementary Figure S5). The results of this analysis were equivocal. PsSiAdV-F remained basal to all avian siadenoviruses, and Gouldian finch adenovirus 1 was basal to the remaining avian siadenoviruses, suggesting that the original observation that adenoviruses infecting passerines first evolved from PsSiAdV-F and that they first evolved in passerines prior to their radiation and colonisation of Europe. However, bootstrap support for the basal nodes in this phylogeny was poor, and further clarification of the evolutionary relationship between the viruses infecting passerine species and the remaining avian siadenoviruses will require that whole genome sequences of adenoviruses derived from Australian passerine species be determined.

## 5. Conclusions

This study reports the first full genome of PsSiAdV-F, which was detected in the tissues of the critically endangered, orange-bellied parrot dying from a bacterial septicemia. The PsSiAdV-F genome recovered in this study is highly divergent and forms a clade that it is basal to all known avian siadenoviruses. Despite being highly divergent, it retains all of the core genes and genes of unknown function found in other members of *Siadenovirus* genus, suggesting that the genetic organisation of the *Siadenovirus* genus is highly stable. Increasing evidence suggests that the clade containing PsSiAdV-F evolved in primitive Australian parrots, which then became the progenitor of adenoviruses in passerine species and subsequently other species of birds. Additional studies screening for PsSiAdV-F in wild Australian parrots and generating whole genome sequences of siadenoviruses of Australian native passerine species will be required to prove this.

## Figures and Tables

**Table 1:** Predicted protein-coding genes of PsSiAdV-F.

PsSiAdV-F Synteny	Start (nt)	Stop (nt)	Strand	Size (aa)	Synteny to PsAdV-D/TAdV-A *	Identity (%)
ORF01 sialidase	108	1970	+	620	Sialidase	40.32
ORF02 hypothetical protein	1983	2312	+	109	hypothetical protein	40.48
ORF03 IVa2	3429	2341	−	362	IVa2 *	67.40
ORF04 DNA polymerase	6742	3419	−	1107	DNA polymerase	60.95
ORF05 pTP	8472	6739	−	577	pTP	59.79
ORF06 52K	8551	9423	+	290	52K	71.76
ORF07 pIIIa	9413	10,909	+	498	pIIIa	60.89
ORF08 penton	10,931	12,271	+	446	Penton	68.78
ORF09 pVII	12,272	12,661	+	129	pVII	66.67
ORF10 pX	12,663	12,839	+	58	pX	67.86
ORF11 pVI	12,857	13,507	+	216	pVI	52.44
ORF12 hexon	13,517	16,285	+	922	Hexon	73.65
ORF13 protease	16,282	16,902	+	206	Protease	60.89
ORF14 early E2	17,984	16,932	−	350	early E2	60.86
ORF15 100K	18,189	20,183	+	664	100K	56.04
ORF16 22K	20,074	20,367	+	97	22K	71.01
ORF17 33K	20,386	20,667	+	93	33K *	70.00
ORF18 pVIII	20,728	21,189	+	153	pVIII	43.30
ORF19 E3	21,137	21,706	+	189	E3	31.82
ORF20 U exon	21,971	21,753	−	72	U exon	42.50
ORF21 fiber protein	21,970	23,001	+	343	fiber protein	35.29
ORF22 hypothetical protein	23,118	23,795	+	225	hypothetical protein	40.10
ORF23 hypothetical protein	24,330	23,806	−	174	hypothetical protein	43.04
ORF24 hypothetical protein	24,578	24,330	−	82	hypothetical protein	42.86
ORF25 hypothetical protein	25,373	24,846	−	175	hypothetical protein	27.38

Notes: PsAdV-D, psittacine siadenovirus D (GenBank accession no. MN687905.1); TAdV-A, turkey siadenovirus A (GenBank accession no. AF074946); PsSiAdV-F, psittacine siadenovirus F (GenBank accession no. MW365934); Asterisks (*) denotes synteny to TAdV-A; aa, amino acid; nt, nucleotide; %, percentage.

**Table 2:** Pairwise identity and comparative G + C (%) content analysis of representative siadenovirus species against PsSiAdV-F based on amino acid sequences of selected core proteins and complete genome sequences. Highest identities are highlighted with bold front.

Siadenovirus	Genome Identity (%)	G + C Content	% Pairwise AA Identities with PsSiAdV-F								
			Sialidase	IVa2	DNA pol	pTP	Penton	Hexon	Protease	DBP	Fiber
Psittacine siadenovirus F [MW365934]		36.9									
Penguin siadenovirus A [KP144329]	53.67	35.6	NA	**66.67**	61.26	60.38	70.02	70.24	61.08	60.97	**23.82**
Turkey siadenovirus A [AC_000016]	54.56	34.9	34.40	**66.67**	60.34	57.17	65.26	69.99	61.58	59.94	19.87
Skua siadenovirus A [HM585353]	**57.06**	34.2	**44.44**	66.58	**61.84**	57.93	69.93	72.22	58.62	**61.43**	19.05
Raptor siadenovirus A [EU715130]	55.61	38.5	39.51	65.11	60.40	59.93	**70.98**	**73.79**	62.56	58.18	18.38
Psittacine siadenovirus E [MK227353]	54.00	37.4	38.16	63.29	58.99	**60.69**	67.86	73.14	**62.75**	58.86	14.06
Psittacine siadenovirus D [MN687905]	31.11	36.9	39.50	64.93	59.98	60.41	68.30	73.51	60.29	60.86	14.72
Psittacine siadenovirus D [MK695679]	54.45	36.9	38.10	64.93	60.25	60.59	68.75	72.76	61.77	60.57	14.78
Frog siadenovirus A [NC_002501]	49.47	37.9	36.96	51.91	51.57	46.65	60.54	67.02	54.68	50.28	20.23

Note: NA—open reading frames are not available in the genome; bold font denotes highest identities values.

## Data Availability

The sequences and associated data analysed in this have been deposited in NCBI GenBank under the accession number MW365934.

## Supplementary Materials

The following are available online at www.mdpi.com/article/10.3390/v13091714/s1, Figure S1: Phylogenetic tree showing the possible evolutionary relationship of novel psittacine siadenovirus F with other selected AdVs. A maximum likelihood (ML) tree was constructed using amino acid sequences of the complete DNA-dependent DNA polymerase gene, Figure S2: Phylogenetic tree showing the possible evolutionary relationship of novel psittacine siadenovirus F with other selected AdVs. A maximum likelihood (ML) tree was constructed using amino acid sequences of the complete pTP gene, Figure S3: Phylogenetic tree showing the possible evolutionary relationship of novel psittacine siadenovirus F with other selected AdVs. A maximum likelihood (ML) tree was constructed using amino acid sequences of the complete penton gene, Figure S4: Phylogenetic tree showing the possible evolutionary relationship of novel psittacine siadenovirus F with other selected AdVs. A maximum likelihood (ML) tree was constructed using amino acid sequences of the complete hexon gene, Figure S5: Phylogenetic tree showing the possible evolutionary relationship of novel psittacine siadenovirus F with other selected AdVs. A maximum likelihood (ML) tree was constructed using amino acid sequences of the partial DNA-dependent DNA polymerase gene.
