# Supplementary material for: Genomic Characterisation of a Highly Divergent Siadenovirus (Psittacine Siadenovirus F) from the Critically Endangered Orange-Bellied Parrot (Neophema chrysogaster)
Source: Viruses. 2021 Aug 28;13(9):1714. doi: 10.3390/v13091714 (PMC8472863; doi:10.3390/v13091714)
Supplement: Supplementary file 1 [file viruses-13-01714-s001.zip › viruses-1339233-supplementary.pdf]

viruses

MDPI

Supplementary File

Genomic Characterisation of a Highly Divergent Siadenovirus (Psittacine Siadenovirus F) from the Critically Endangered Orange-Bellied Parrot (*Neophema Chrysogaster*)

Ajani Athukorala, David N. Phalen, Ashutosh Das, Karla J. Helbig, Jade K. Forwood and Subir Sarker

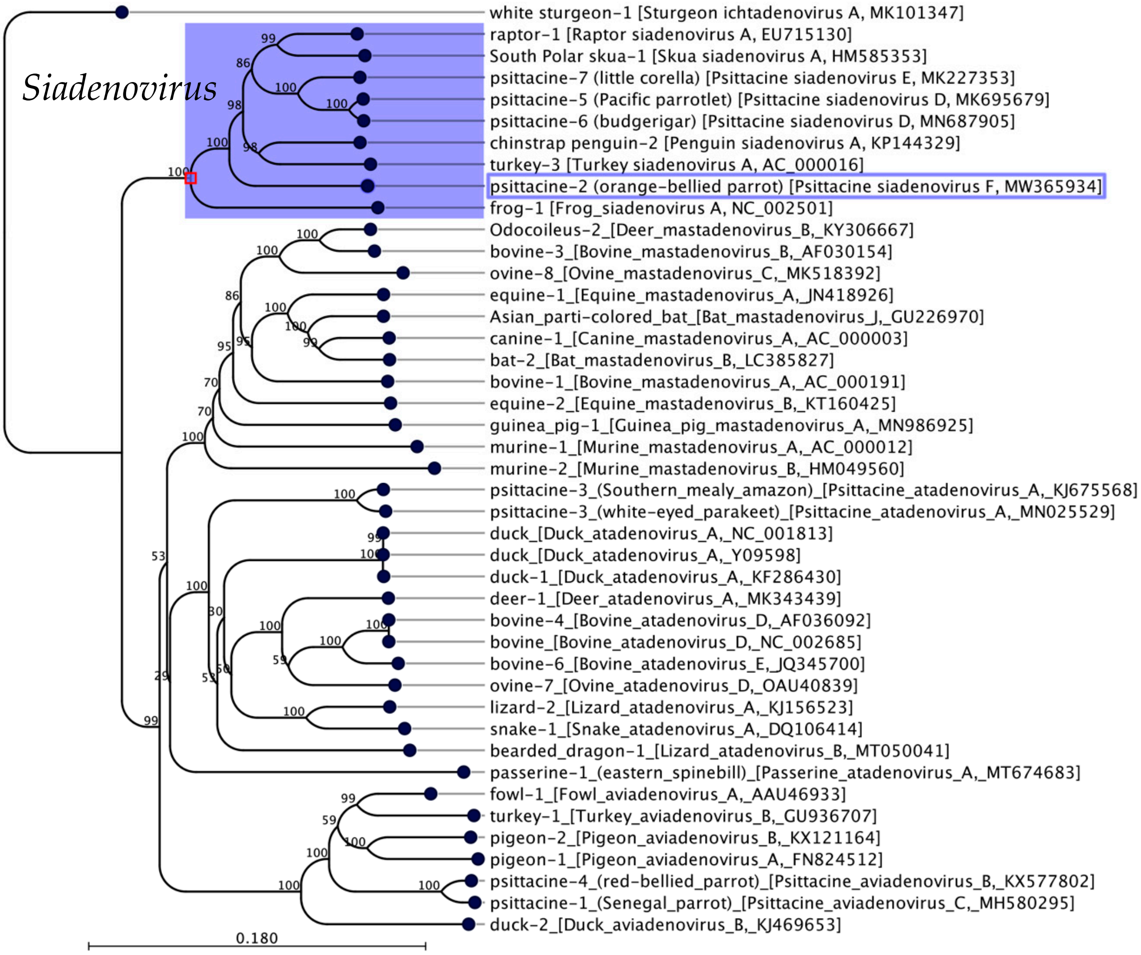

Figure S1. Phylogenetic tree showed the possible evolutionary relationship of novel psittacine siadenovirus F with other selected AdVs. Maximum likelihood (ML) tree was constructed using amino acid sequences of the complete DNA-dependent DNA polymerase gene. Selected protein sequences were aligned with MAFTT (version 7.45) [33] in Geneious (version 10.2, Biomatters, Ltd., Auckland, New Zealand) under the BLOSUM62 scoring matrix and gap open penalty =1.53. The unrooted ML tree was constructed under the WAG substitution model, and 1000 bootstrap replicates using tools available in CLC Genomics Workbench (version 9.5.4, CLC bio, a QIAGEN Company, Prismet, Aarhus C, Denmark). The numbers on the left shown bootstrap values as percentages and the labels at branch tips refer to original host species followed by AdVs name and GenBank accession number in parentheses. The novel psittacine siadenovirus F was shown in purple colour box.

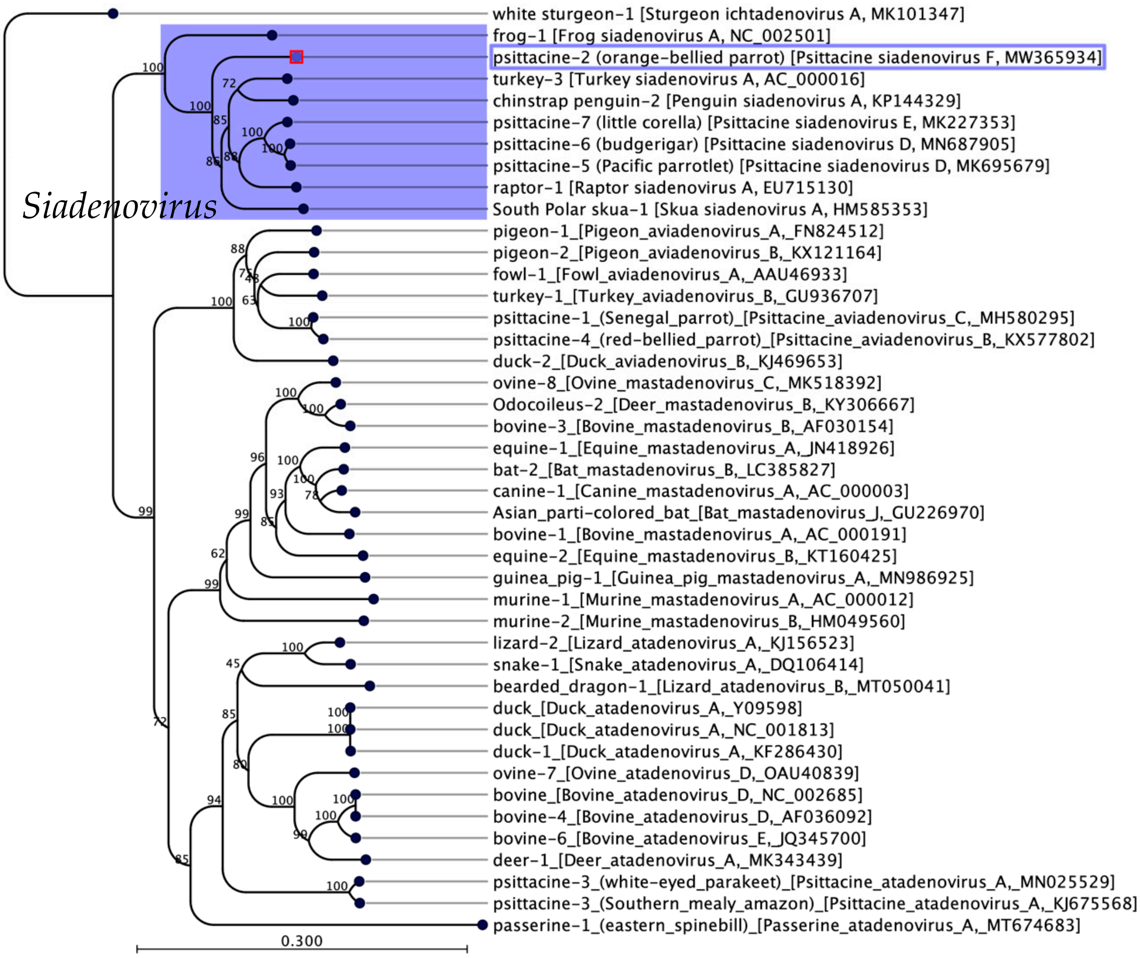

Figure S2. Phylogenetic tree showed the possible evolutionary relationship of novel psittacine siadenovirus F with other selected AdVs. Maximum likelihood (ML) tree was constructed using amino acid sequences of the complete pTP gene. Selected protein sequences were aligned with MAFTT (version 7.450) [33] in Geneious (version 10.2.2, Biomatters, Ltd., Auckland, New Zealand) under the BLOSUM62 scoring matrix and gap open penalty =1.53. The unrooted ML tree was constructed under the WAG substitution model, and 1000 bootstrap replicates using tools available in CLC Genomics Workbench (version 9.5.4, CLC bio, a QIAGEN Company, Prismet, Aarhus C, Denmark). The numbers on the left shown bootstrap values as percentages and the labels at branch tips refer to original host species followed by AdVs name and GenBank accession number in parentheses. The novel psittacine siadenovirus F was shown in purple colour box.

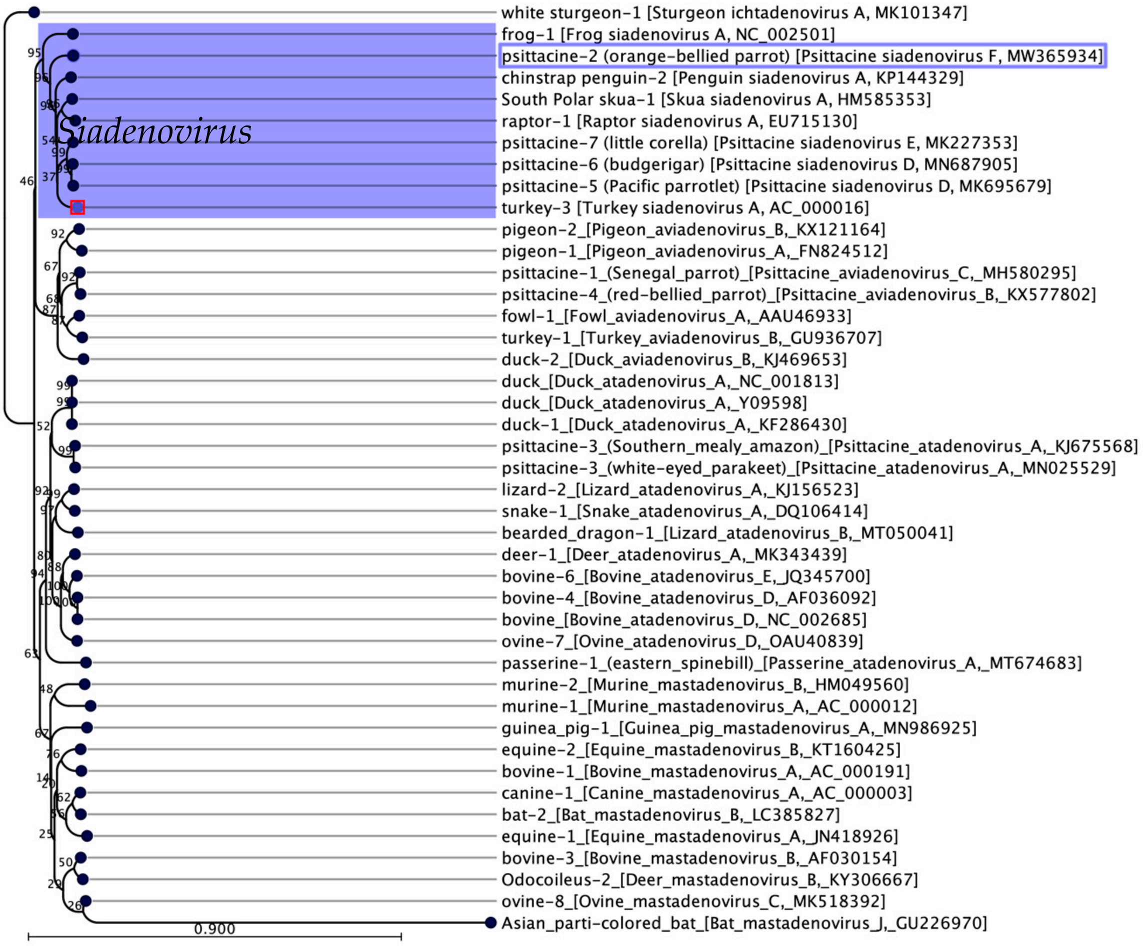

Figure S3. Phylogenetic tree showed the possible evolutionary relationship of novel psittacine siadenovirus F with other selected AdVs. Maximum likelihood (ML) tree was constructed using amino acid sequences of the complete penton gene. Selected protein sequences were aligned with MAFTT (version 7.450) [33] in Geneious (version 10.2.2, Biomatters, Ltd., Auckland, New Zealand) under the BLOSUM62 scoring matrix and gap open penalty =1.53. The unrooted ML tree was constructed under the WAG substitution model, and 1000 bootstrap replicates using tools available in CLC Genomics Workbench (version 9.5.4, CLC bio, a QIAGEN Company, Prismet, Aarhus C, Denmark). The numbers on the left shown bootstrap values as percentages and the labels at branch tips refer to original host species followed by AdVs name and GenBank accession number in parentheses. The novel psittacine siadenovirus F was shown in purple colour box.

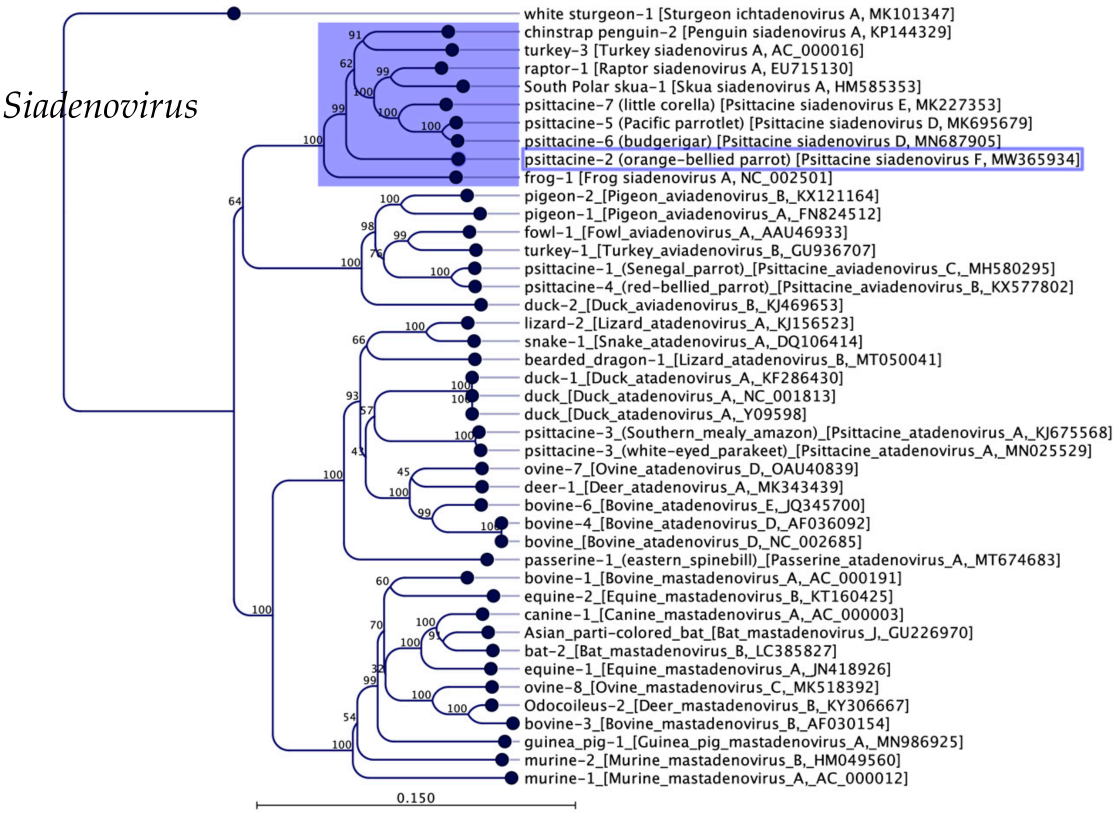

Figure S4. Phylogenetic tree showed the possible evolutionary relationship of novel psittacine siadenovirus F with other selected AdVs. Maximum likelihood (ML) tree was constructed using amino acid sequences of the complete hexon gene. Selected protein sequences were aligned with MAFTT (version 7.450) [33] in Geneious (version 10.2.2, Biomatters, Ltd., Auckland, New Zealand) under the BLOSUM62 scoring matrix and gap open penalty =1.53. The unrooted ML tree was constructed under the WAG substitution model, and 1000 bootstrap replicates using tools available in CLC Genomics Workbench (version 9.5.4, CLC bio, a QIAGEN Company, Prismet, Aarhus C, Denmark). The numbers on the left shown bootstrap values as percentages and the labels at branch tips refer to original host species followed by AdVs name and GenBank accession number in parentheses. The novel psittacine siadenovirus F was shown in purple colour box.

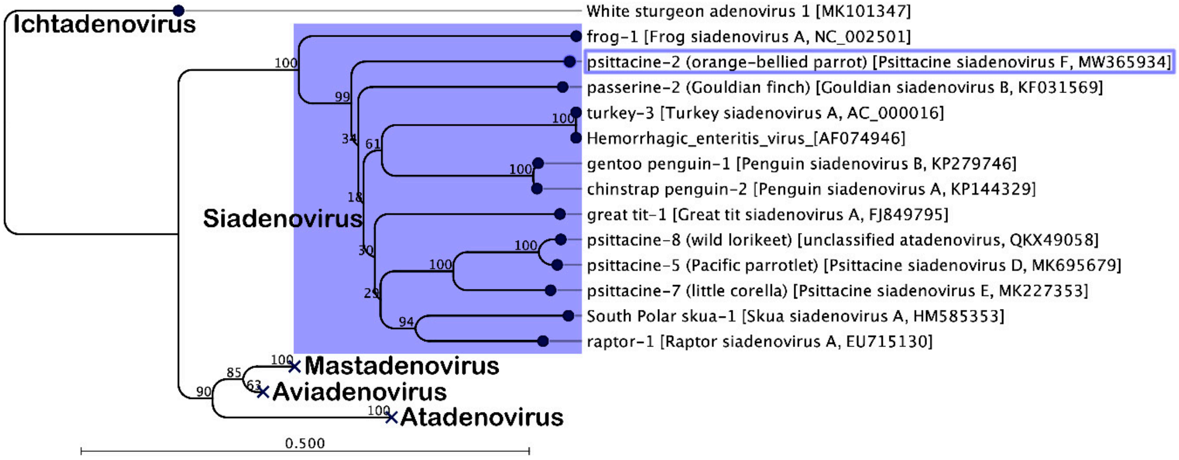

Figure S5. Phylogenetic tree showed the possible evolutionary relationship of novel psittacine siadenovirus F with other selected AdVs. Maximum likelihood (ML) tree was constructed using amino acid sequences of the partial DNA-dependent DNA polymerase gene. Selected protein sequences were aligned with MAFTT (version 7.450) [33] in Geneious (version 10.2.2, Biomatters, Ltd., Auckland, New Zealand) under the BLOSUM62 scoring matrix and gap open penalty =1.53. The unrooted ML tree was constructed under the WAG substitution model, and 1000 bootstrap replicates using tools available in CLC Genomics Workbench (version 9.5.4, CLC bio, a QIAGEN Company, Prismet, Aarhus C, Denmark). The numbers on the left shown bootstrap values as percentages and the labels at branch tips refer to original host species followed by

AdVs name and GenBank accession number in parentheses. The novel psittacine siadenovirus F was shown in purple colour box. Clades relevant to the genera *Mastadenovirus, Aviadenovirus* and *Atadenovirus* are collapsed.
